# Translation of evidence-based Assistive Technologies into stroke rehabilitation: users’ perceptions of the barriers and opportunities

**DOI:** 10.1186/1472-6963-14-124

**Published:** 2014-03-12

**Authors:** Ann-Marie Hughes, Jane Helena Burridge, Sara Holtum Demain, Caroline Ellis-Hill, Claire Meagher, Lisa Tedesco-Triccas, Ruth Turk, Ian Swain

**Affiliations:** 1 Faculty of Health Sciences, University of Southampton, Southampton, UK; 2 Electronics and Computer Sciences, Faculty of Physical & Applied Sciences, University of Southampton, Southampton, UK; 3 School of Health and Social Care, Bournemouth University, Bournemouth, UK; 4 School of Design, Engineering and Computing, Bournemouth University, Bournemouth, UK; 5 Clinical Science and Engineering, Salisbury NHS Foundation Trust, Salisbury, UK

**Keywords:** Assistive technology, Upper limb, Stroke rehabilitation, Translation into practice, Perceptions

## Abstract

**Background:**

Assistive Technologies (ATs), defined as “electrical or mechanical devices designed to help people recover movement”, demonstrate clinical benefits in upper limb stroke rehabilitation; however translation into clinical practice is poor. Uptake is dependent on a complex relationship between all stakeholders. Our aim was to understand patients’, carers’ (P&Cs) and healthcare professionals’ (HCPs) experience and views of upper limb rehabilitation and ATs, to identify barriers and opportunities critical to the effective translation of ATs into clinical practice. This work was conducted in the UK, which has a state funded healthcare system, but the findings have relevance to all healthcare systems.

**Methods:**

Two structurally comparable questionnaires, one for P&Cs and one for HCPs, were designed, piloted and completed anonymously. Wide distribution of the questionnaires provided data from HCPs with experience of stroke rehabilitation and P&Cs who had experience of stroke. Questionnaires were designed based on themes identified from four focus groups held with HCPs and P&Cs and piloted with a sample of HCPs (N = 24) and P&Cs (N = 8). Eight of whom (four HCPs and four P&Cs) had been involved in the development.

**Results:**

292 HCPs and 123 P&Cs questionnaires were analysed. 120 (41%) of HCP and 79 (64%) of P&C respondents had never used ATs. Most views were common to both groups, citing lack of information and access to ATs as the main reasons for not using them. Both HCPs (N = 53 [34%]) and P&C (N = 21 [47%]) cited Functional Electrical Stimulation (FES) as the most frequently used AT. Research evidence was rated by HCPs as the most important factor in the design of an ideal technology, yet ATs they used or prescribed were not supported by research evidence. P&Cs rated ease of set-up and comfort more highly.

**Conclusion:**

Key barriers to translation of ATs into clinical practice are lack of knowledge, education, awareness and access. Perceptions about arm rehabilitation post-stroke are similar between HCPs and P&Cs. Based on our findings, improvements in AT design, pragmatic clinical evaluation, better knowledge and awareness and improvement in provision of services will contribute to better and cost-effective upper limb stroke rehabilitation.

## Background

Stroke is a leading cause of disability world-wide [1]. Because it is an age related disease, incidence is likely to rise. Prevalence is also likely to rise due to better survival rates and long-term care. In the UK alone over 110,000 people have a stroke annually and over 300,000 people are living with disability as a result of a stroke. The combined societal cost of treatment and lost productivity is £8.9 billion a year, with treatment costs accounting for approximately 5% of total UK National Health Service (NHS) costs [2].

Disability and dependence associated with reduced upper limb function following stroke impacts on patients’ and carers’ (P&Cs’) quality of life [3] and national economies [4,5]. Approximately 85% of stroke patients have upper limb impairments at stroke onset. The majority of these will not regain useful function [6-8], particularly affecting the 75% of younger individuals who want to return to work [9]. Consequently evaluation of the effectiveness of rehabilitation interventions after the acute phase of stroke is one of the National Stroke Strategy’s top ten priorities for stroke services research, and identification of best practice in the rehabilitation of the upper limb in patients with stroke with respect to timing, content and dosage is the highest prioritised research topic [10]. Demand for more cost-effective treatment is leading to changes in practice that are likely to involve Assistive Technologies (ATs) in addition to conventional occupational therapy and physiotherapy. National strategies and frameworks continue to emphasise the need for informed decision making in healthcare that are research led and evidence-based, yet the UK, Australian and US National Clinical Guidelines for Stroke indicate that there is limited research to assess efficacy of ATs, either individually or in combination [8,11,12].

It has been demonstrated that the main predictor of success in physical therapy is intensity [13] and that key components are optimum content, optimum delivery and optimum structure [5]. ATs may provide increased intensity without a corresponding increase in clinical contact time, motivating and relevant activities (either functional or impairment based), and may be performed outside the hospital [14]. They therefore have the potential to improve cost effectiveness of upper limb stroke rehabilitation.

### Rehabilitation

An important aim of rehabilitation is to promote neuroplasticity within the central nervous system and ameliorate secondary effects of stroke such as muscle weakness and reduced range of motion. Neurological re-organisation or learning occurs in response to experience gained through afferent inputs. Studies have demonstrated that functions, previously the responsibility of damaged areas of the cortex, may be taken over by adjacent areas or by areas in the contralateral hemisphere [15]. However, the disability itself can be a significant barrier to obtaining the afferent feedback necessary to promote learning. The aim of ATs is therefore promotion of these processes, enabling more sustained and engaging practice i.e. supporting behavioural training. There is growing evidence that relearning of function is encouraged by appropriate functional practice of meaningful tasks aimed at acquiring a practical skill, rather than simple repetition of a movement [16]. In the future, ATs are likely to be used by patients at home, used either independently or supported by the multidisciplinary team, which will change the focus of responsibility for rehabilitation and may result in the long term continuation of regular exercise and functional training.

### Assistive Technologies (ATs)

The definition of Assistive Technologies (ATs) used in this study was electrical or mechanical devices designed to help people recover movement of their upper limb. ATs are increasingly used to augment conventional physiotherapy and occupational therapy. Descriptions used to explain the ATs mentioned in the questionnaires (Additional files 1 and 2) are as follows: Virtual Reality (VR), computer games that you play by moving your arm and hand. Sometimes you see an image of your arm and the way it is moving on the computer screen; Dynamic splints: e.g. Saeboflex - devices that you wear. They may have strapping or springs to help you open your hand to pick up objects. Biofeedback: where the device tells you immediately about your arm movement; Robots: devices that support your arm and/or hand and help you to move while you either play computer games or practise manual tasks; Constraint Induced Movement Therapy (CIMT): where you wear a glove on your good hand for about six hours a day for a few weeks; and Functional Electrical Stimulation (FES): sticky pads or a device over your skin which transmits small electrical impulses.

Evidence is growing for the use of ATs to reduce impairments and in some cases improve function. Systematic reviews support the use of robotics [17], FES [18], CIMT [19], and VR [20] and in combination [21-26]. Although studies of the combined treatment have demonstrated benefits, in general, they have been inadequately powered. Despite the inconclusive clinical evidence for ATs, rapid advances continue to be made in the technology. It is therefore critical that design is informed not only by trials of clinical effectiveness, most of which have been experimental rather than pragmatic, but also by what is acceptable to users.

Design of clinically useful ATs requires consideration of multiple and sometimes competing factors such as effectiveness, cost, ease of use and aesthetics and, therefore, requires knowledge of users’ priorities. There has been speculation over possible reasons for the lack of translation of ATs into clinical practice, but limited empirical research.

Our aim was to understand P&Cs’ and healthcare professionals’ (HCPs’) experiences and perceptions of ATs and upper limb stroke rehabilitation, to identify barriers and opportunities critical to the effective translation of ATs into clinical practice. To achieve our aim we sought the views of P&Cs and HCPs via questionnaires. The design of the questionnaires was informed by output from an interactive exhibition and focus groups. Two hundred and four people (HCP 49%, P&C 51%) attended the interactive exhibition over 3 days with 12 different companies displaying 27 different ATs. Awareness was raised about the research study, and people were invited to leave contact details if they wanted to have further information about the study. The exhibition thus provided an opportunity for people to reflect on their own experiences and needs in the context of a range of ATs and identified a group of people with an interest in this area. This group was sampled to form the four focus groups of HCPs and P&Cs who had and had not used ATs. Full details of the focus group methodology, results and conclusions are available [27].

## Method

HCP and P&C questionnaire design and development, piloting, dissemination and data analysis plan is described. The questionnaires (Additional files 1 and 2) were co-designed and piloted, using iterative testing and revising throughout the process.

### Participants

24 HCPs and 8 P&Cs were recruited to contribute to the design and piloting of the questionnaires. All were representative of the questionnaire target groups. Criteria for recruitment were, for the HCP group, at least one of: a) member of the project steering committee and/or researcher on the project; b) attendee at the exhibition; c) HCP working in stroke rehabilitation. All P&Cs were either stroke survivors or carers of a stroke survivor, with or without experience of ATs, who had: a) had attended of the exhibition; or b) been a member of one of the focus groups; or c) was a member of a local stroke group.

Questionnaire respondents were self-selected. Responses to questions 1 and 2 in the HCP questionnaire ensured that respondents satisfied the single criterion: were either a nurse, occupational therapist, physiotherapist, doctor or other HCP who had experience of upper limb stroke rehabilitation. Response to question 2 in the P&C questionnaire ensured that respondents satisfied the criteria: were either a person who has had a stroke or a carer, such as a friend, or relative of someone who has had a stroke.

### Questionnaire design and development

Questions were identified from the following sources: focus group data; feedback from the exhibition and published systematic reviews of the AT literature [17,19,28]. Structure and design of the questionnaires was informed by published literature on questionnaire design [29-31]. The authors reached a consensus on content and design. Revisions to the first drafts of the questionnaires were then made following consultation with the project steering committee (N = 9) comprising national and international experts in: clinical services; clinical, engineering and psychology AT research; health economics as well as patient group representatives (Stroke Association, Different Strokes), and a stroke survivor. This ensured that questions on all aspects of ATs were included in the questionnaires and that the questions were generated from the perspective of the target audience. Initial face and content validity were established through consultation with experts within the steering committee.

The design of the two questionnaires (Additional files 1 and 2) was kept as similar as possible so that comparisons could be made between the two groups of respondents. Within the questionnaires, open questions were avoided where possible to allow direct coding. Closed questions included: Yes/No answers, Likert scales and multiple-choice. Multiple-choice questions were used to identify which ATs were used. Choices had been identified from focus group data and were: FES, VR, CIMT; Dynamic Splints; biofeedback; rehabilitation robots and an option to identify ‘any other’ that was not listed.

### Questionnaire piloting

The questionnaires were piloted with four HCPs and four P&Cs who had attended the focus groups or the exhibition and 20 HCPs, researchers and patient representatives (recruited from the steering committee and from HCPs working in stroke rehabilitation) and four P&C (from a local stroke club) who had not attended and had not had input into the design. Including naïve participants reduced bias towards the views of participants who had contributed to the initial development. All participants were asked for their comments on the scope of the topics and whether there were missing areas of interest. Based on participant responses, amendments were made to the wording of some questions and the omission of two questions. Following revision, two further samples of P&Cs and HCPs who had attended the focus groups were asked to complete the questionnaires independently, after which they took part in a telephone discussion with one of the researchers to check that all questions conveyed the same meaning to each participant and were not ambiguous. Participants were also asked to comment on the style and ease of completion etc.

Following further minor revisions, online versions of the questionnaires were developed from the paper versions. Question order was the same in both versions to reduce risk of bias by primacy effects. The electronic versions of the questionnaires were then piloted with representatives of each target population who had not attended the focus groups (N = 2 HCPs N = 2 P&Cs).

During development and piloting, user feedback identified issues with suitability and utility within the respondent groups and, in response, modifications were made to content, style and language.

### Questionnaire dissemination

The HCP questionnaires were advertised widely through HCP special interest groups, at professional conferences, via professional bodies, through the project website hosted by the UK National Institute of Health Research and through personal contacts. Preliminary questions about profession and experience provided demographic participant data and were used to screen respondents to ensure they satisfied the selection criteria. However, as the questionnaires were predominantly online, whilst we targeted the relevant groups, we were unable to control who would respond or the veracity of their responses. For the P&C questionnaires a pre-awareness campaign was launched via UK charities: Different Strokes (Newsletter) [32] and the websites of The Stroke Association and Different Strokes, and then also distributed via voluntary organisations representing P&Cs. Preliminary questions provided demographic participant data and ensured that respondents satisfied the selection criteria. To ensure maximum inclusivity, the questionnaires could also be completed by telephone, online via a hyperlink on an email, and paper based via post.

### Ethical approval and consent

Ethical approval was granted from the Isle of Wight, Portsmouth and South East Hampshire Research Ethics Committee (09/H0501/71). In accordance with ethical approval, consent to participate was assumed following completion and submission of the questionnaires. Responses were anonymous.

### Analysis

Returned questionnaires were included in the analysis if questions 1–3 had been completed. Nominal, discrete and ordinal data from the online surveys populated an Excel spreadsheet. Double data entry was used to input the results from the paper-based copies. The resulting data were then imported into SPSS (version 18) for statistical analysis. Categorical data (e.g. gender, profession and carer or patient) and responses to “have you used any of the following ATs?” were described with counts and percentages. Factors affecting ‘ideal design’ were ranked using an ordinal scale. Descriptive, rather than inferential data analysis was used to summarise data. Sub-group analysis, by for example profession or experience, was not performed due to the limitations of sample size.

## Results

A total of 419 questionnaires were returned, 296 (71%) from HCPs and 123 (29%) from P&Cs. Of the 296 HCP returns, 26 of the 44 (59%) were posted questionnaires and 270 of the 635 (43%) were online accessed questionnaires. Of the 123 P&C returns, 54 (44%) were postal and, although 597 people opened the online version, only 59 (10%) were completed beyond question 3 and therefore included in the analysis. Ten P&C questionnaires were completed face-to-face. Data from all modes of response were combined. Four people who responded to the HCP questionnaire were not included in the analysis as they did not satisfy the selection criteria being either Speech and Language therapists (N = 2), so did not have experience of upper limb stroke rehabilitation, or were not HCP according to our definition (N = 2). Not all respondents answered all questions; therefore the numbers of respondents are presented for each question.

### Demographic data

Returned questionnaires from 292 HCPs (43% Occupational therapists (OT), 51% Physiotherapists (PT), 5% Nurses and 1% Doctors) were analysed. The mean number of years’ experience of working with stroke was 10.9 (SD = 7.7 min-max 1–45). HCPs respondents (N = 288) worked across different and sometimes multiple settings: acute (N = 152), subacute (N = 233), chronic (N =9 6), and private practice (N = 15). Returned questionnaires from 123 P&Cs were analysed; 99 (80%) were patients and 24 (20%) were carers. Thirteen carers completed the questionnaire on behalf of a patient. Mean time post stroke (patients) was 8 years (SD 8.4, min-max 0–73).

### Access to information about ATs

Most HCPs (92%) had accessed information on ATs (265/288). Sources were, in order: Continuing Professional Development courses (176); other non-Continuing Professional Development courses (171); Internet (152); through AT companies (88); through their hospital (81); undergraduate courses (13); and other sources (37) which were mainly conferences (17) and colleagues (10). Just over half reported accessing information from more than two sources (145/288). A smaller proportion of P&C (41%) had accessed information on ATs (51/123). Sources were, in order: Internet (29); through their hospital (28); through AT companies (12); taking part in training courses for therapists (3); and other, which were mainly stroke groups (31). 26 respondents had accessed information from more than two sources and three had gained information from three or more sources.

### Experience of using of ATs

Both HCP and P&C were asked if and how much they used ATs and to cite their reasons for not using them (Table 1). Over half, 59% (170/290) of HCPs had used ATs in their clinical practice and under half, 44% (108/122) of P&Cs had used them.

**Table 1:** HCP and P&C experience of using ATs, and reasons for not using ATs

**Used ATs**	**HCPs (N = 290) (%)**	**P&C (N = 122) (%)**
Often	23 (8)	14 (11)
Sometimes	147 (51)	30 (25)
Never	120 (41)	78 (64)
	No reason given by 6 respondents	Reasons given by 78 respondents were:
	Reasons given by 114 respondents were:	Lack of knowledge (N = 48)
	Lack of access (N = 98)	Lack of access (N = 21)
	Lack of knowledge (N = 27)	I do not think I need one (N = 9)
	Do not think they work (N = 1)	A professional told me they were not appropriate (N = 4)
	12/114 cited 2 reasons	4/78 cited 2 reasons

### Which ATs are used or prescribed?

**Figure 1:**
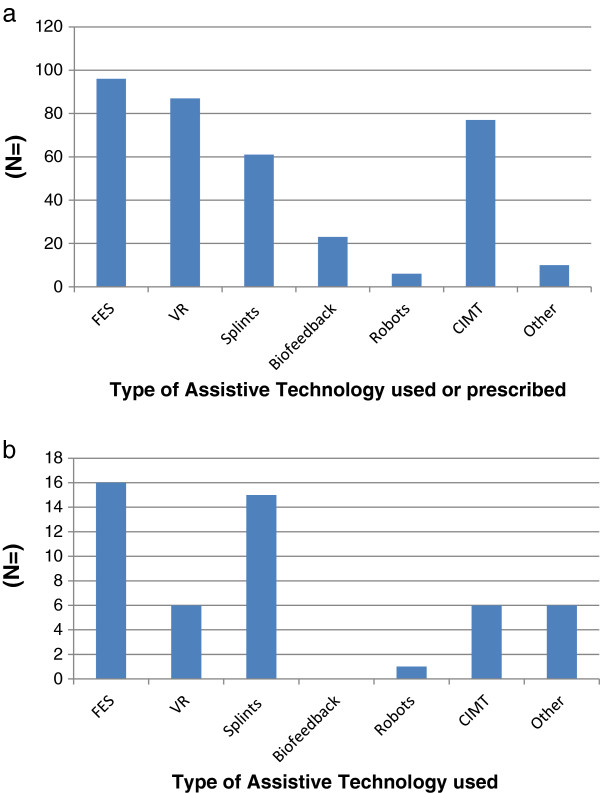
**Responses to the question ‘which of the following ATs have you ever used (or prescribed)’? (a)** HCPs **(b)** P&Cs.

154 HCPs and 45 P&Cs provided information on the ATs they used or prescribed, or in the case of P&C used, and this is presented in Figure 1a and b respectively. 64 HCPs reported using more than two ATs and five P&Cs used two types of AT.

### Which ATs are used most frequently?

**Figure 2:**
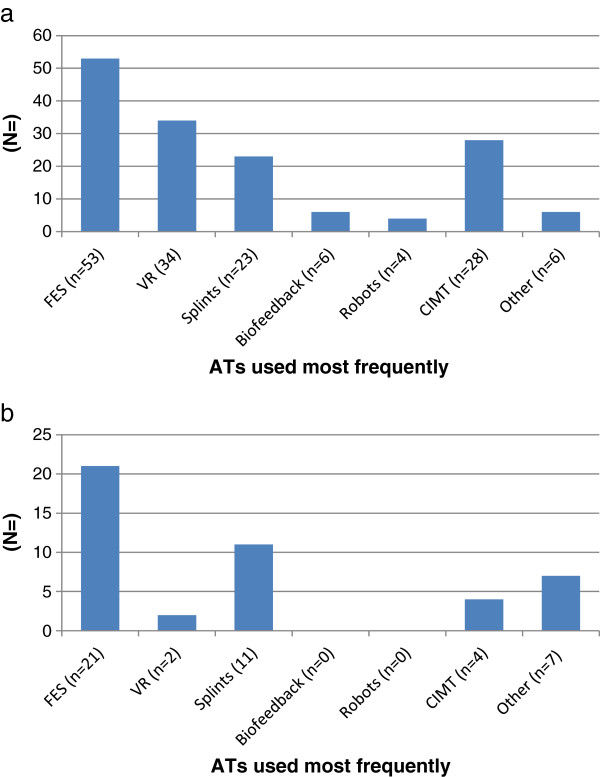
**Responses to the question ‘which AT do you use (or prescribe) most frequently? (a)** HCPs **(b)** P&Cs.

HCPs and P&Cs were asked to report what AT they used most frequently. The results are demonstrated in Figure 2a and b respectively. The ATs most frequently used by HCPs (34%) and P&Cs were FES (47%) and the least used by both groups were robot therapy and biofeedback.

### Respondents’ views on the ATs they had used most frequently

Respondents who reported using or prescribing a specific AT frequently (154 HCPs and 45 P&Cs) were asked the question: ‘Thinking about the AT you use most often, do you think this device is….’. The responses are summarised for the HCPs (Table 2) and P&Cs (Table 3). The number of responders who cited each AT as that most frequently used is shown and varied among HCPs from N = 52 for VR to N = 4 for robots. None of the P&Cs included Robots or Biofeedback as the AT they used most frequently. Not all responders answered each question; therefore the number of responders is also given. Positive responses out of the total number of responses to each question are presented as a percentage. These ‘popular’ ATs were easy to set up, durable, comfortable, low risk, evidence-based, good value, and suitable for home use, and therefore could mainly be used outside therapy sessions. In general, therapists did not think that even these popular ATs ‘looked good’ or ‘were fun’ for patients to use. However, they were more likely to be used at home than in therapy sessions. P&Cs did not think that ATs were good value for money.

**Table 2:** HCPs responses “yes” to the Question: ‘Thinking about the AT you use most often, do you think this device is…’

**‘Thinking about the AT you use most often, do you think this device is…**	**VR N = 34 (%)**	**Dynamic Splint N = 23 (%)**	**Biofeedback N = 6 (%)**	**Robots N = 4 (%)**	**CIMT N = 28 (%)**	**FES N = 52 (%)**
Easy set up (N = 154)	31 (91)	10 (43)	3 (50)	2 (50)	23 (82)	39 (75)
Primarily used in therapy sessions (N = 152)	9 (26)	13 (59)	3 (50)	1 (25)	7 (25)	15 (29)
Suitable for home-use (N = 151)	28 (82)	21 (95)	1 (17)	0 (0)	25 (89)	45 (87)
Looks good (N = 149)	28 (82)	8 (35)	2 (40)	3 (75)	5 (19)	12 (24)
Durable and reliable (N = 150)	29 (85)	17 (74)	4 (67)	4 (100)	20 (70)	45 (86)
Comfortable (N = 154)	32 (94)	18 (78)	5 (83)	3 (75)	20 (70)	40 (77)
Safe (N = 151)	30 (88)	17 (74)	6 (100)	4 (100)	26 (93)	51 (98)
Evidence-based (N = 154)	15 (44)	13 (57)	4 (67)	4 (100)	28 (100)	49 (94)
Good value for money (N = 154)	23 (68)	10 (43)	2 (33)	1 (25)	26 (93)	35 (68)
Fun to use (N = 154)	33 (97)	9 (39)	3 (50)	4 (100)	4 (14)	14 (26)

**Table 3:** P&Cs responses “yes” to the Question: ‘Thinking about the AT you use most often, do you think this device is…’

**‘Thinking about the AT you use most often, do you think this device is…**	**VR N = 2 (%)**	**Dynamic splint N = 11 (%)**	**CIMT N = 4 (%)**	**FES N = 21 (%)**
Easy set up (N = 45)	1 (50)	6 (55)	4 (100)	12 (57)
Primarily used in therapy sessions (N = 44)	2 (100)	6 (55)	1 (25)	11 (52)
Suitable for home-use (N = 45)	0 (0)	8 (73)	4 (100)	17 (81)
Looks good (N = 37)	0 (0)	1 (9)	0 (0)	6 (29)
Durable and reliable (N = 41)	0 (0)	5 (45)	2 (50)	13 (62)
Comfortable (N = 42)	2 (100)	6 (55)	3 (75)	13 (62)
Safe (N = 44)	1 (50)	8 (73)	3 (75)	16 (76)
Based on research evidence (N = 42)	2 (100)	3 (27)	2 (50)	15 (71)
Good value for money (N = 43)	0 (0)	4 (36)	2 (50)	5 (24)
Fun to use (N = 41)	1 (50)	4 (36)	1 (25)	5 (24)

### What factors are important in the design of the ideal AT?

The most important factors for the design of an AT were identified by the focus groups. In the questionnaire respondents were asked to rank these ‘important factors’ in order of importance by assigning them a score from one to ten. Their responses are shown in Table 4.

**Table 4:** Ranking of factors identified by both HCPs and P&Cs for design of an ideal AT

**Ranking factors for design of an ideal AT**	**HPC rank**	**P&C rank**
Good research evidence	1	5
Easy to set up and use	2	1
Low risk of harm	3	3
Comfortable	4	2
Durable and reliable (does not breakdown)	5	4
Value for money	6	8
For use mainly unsupervised at home	7	7
Fun	8	6
For use mainly under supervision of a HCP	9	9
Attractive appearance	10	10

(Responses were weighted so that the factors identified as most important were scored 1 and those of least importance scored 10).

### Perceptions about current upper limb rehabilitation and the use of ATs

**Figure 3:**
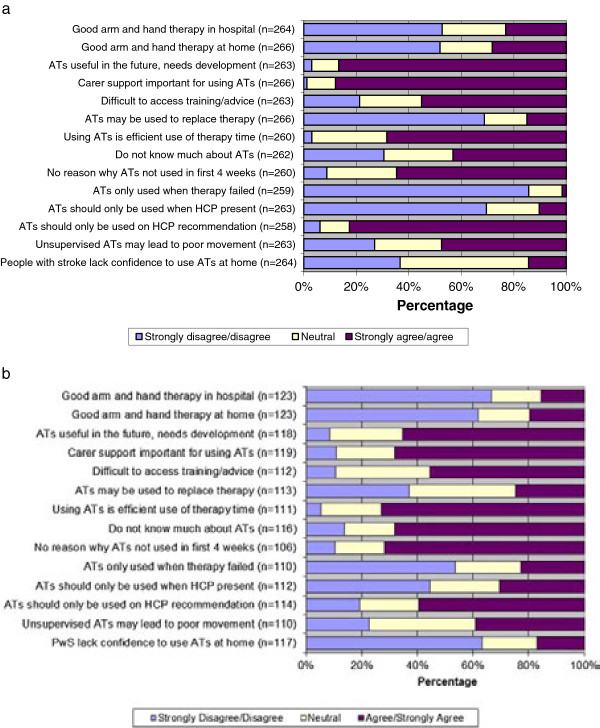
Perceptions about current upper limb therapy and the use of ATs (a) HCPs (b) P&Cs.

It is generally accepted that ATs, if used in upper limb rehabilitation following stroke, will augment conventional treatment and this assumption was confirmed by our respondents. It was therefore also important to gauge satisfaction with current service provision. Respondents’ perceptions about current upper limb therapy and the use of ATs are displayed in Figure 3a and b respectively. Less than 25% of HCPs (Figure 3a) and 20% of P&Cs (Figure 3b) agreed that people had good arm and hand therapy in hospital and at home. This perception underpins our exploration into ways in which satisfaction with therapy can be improved and specifically whether ATs may be useful from the perspective of users.

HCPs and P&Cs were asked the same questions concerning their beliefs in terms of ATs for upper limb therapy. They were presented with a series of statements, generated from the focus group data, and asked whether they strongly agreed, agreed, were neutral, disagreed or strongly disagreed with each statement. The responses are presented below in a simplified form to give three categories (Figure 3a and b). In summary, the views of HCPs and P&Cs were very similar, although P&Cs reported having less knowledge about ATs, had more confidence in using ATs at home than HCPs thought they had and disagreed less strongly that ATs might replace therapy. All respondents viewed using ATs as an efficient use of therapy time and resources; they saw no reasons why ATs should not be used in the first 4 weeks, and disagreed that ATs should only be used when conventional therapy had failed. They did not agree that ATs should only be used with HCPs present, however they had mixed views on whether unsupervised use of ATs would lead to poor movement patterns. They believed that ATs should only be used on HCP recommendation. Both HCPs and P&Cs believed that carer support was important. Respondents believed there was a role for ATs in the future, but that they needed more development.

## Discussion

This survey provides evidence of HCPs and P&Cs’ views on the use of ATs in upper limb stroke rehabilitation and a benchmark from which changing attitudes can be measured. The results are relevant to all healthcare systems and particularly those that are free at the point-of-care such as the UK NHS. ATs will become increasingly important in the drive to deliver cost-effective improvements in stroke rehabilitation outcomes and to satisfy, for example, the UK National Clinical Guidelines for Stroke [8] that state: “Patients who have some arm movement should be given every opportunity to practise activities within their capacity.” Despite the increasing reference to ATs in healthcare policy, research into effectiveness and investment in commercial development, no previous survey has sampled or compared HCPs’ and P&Cs’ views of ATs. Our survey has generated new information about factors that influence clinical use of ATs and the opportunities for and barriers to translation of ATs into clinical practice. Based on our findings, we discuss how opportunities can be exploited and barriers overcome. We also discuss the influence our sample may have had on our findings, the strengths and limitations of the survey and how it will impact on future work.

### Factors that determine clinical use

Strength of evidence for clinical effectiveness and usability has been cited as an important factor influencing translation of rehabilitation research into clinical practice [33,34]. The results of our survey generated evidence to support this: HCPs cited evidence-base as the most important of ten factors for an ideal technology compared to P&Cs who ranked it 5^th^; and who considered both ease of set-up and comfort as more important. However, despite ranking evidence-base as the most important factor, many HCPs prescribed and used ATs that were not evidence-based. FES for example was cited as the most commonly prescribed AT by both HCPs (96/152 [63%]) and P&Cs, (16/45 [36%]), yet research evidence is equivocal. The Evidenced-Based Research in Stroke Rehabilitation, EBRSR) [35], considers the evidence for FES in improving upper extremity function in acute stroke to be strong (level 1a), but the UK National Institute for Clinical Excellence (NICE) and the National Clinical Guidelines for Stroke [8], do not consider it strong enough to recommend that FES is used other than in the context of a clinical trial. VR, the second most prescribed AT by HCPs (87/152 [57%]), currently has little evidence for its benefit, and is not recommended by any guidelines, but HCPs scored it highly on being ‘fun’ and ‘easy’ to use. Robot therapy, the least cited technology used by either HCPs or P&Cs, by comparison receives a more positive recommendation from both the EBRSR and the UK National Guidelines in terms of evidence for reduction of impairment and improvement of shoulder and elbow function which state that: Robot-assisted movement therapy should only be used as an adjunct to conventional therapy when the goal is to reduce arm impairment or in the context of a clinical trial. CIMT, for which there is strong (level 1a) research evidence and which is recommended by both EBRSR and the UK Stroke Guidelines was cited as prescribed by HCPs 77/152 [51%] but the AT most frequently prescribed by only 28/154 (18%) of HCPs. For P&C, CIMT was cited as the most frequently used AT by 4/45 [<10%] of P&Cs who responded.

Conflict between clinical use and research evidence may reflect the absence of definitive research evidence for any ATs. Other reasons for why some ATs are not more widely used could be limited applicability – CIMT is only suitable for people with >20 degrees of wrist extension and 10 degrees of finger extension, or the unwillingness of HCPs or P&Cs to use it for practical reasons, or, in the case of robots and biofeedback, factors such as cost, unsuitability for home-use, or use without supervision. In summary therefore, research evidence and evidence-based recommendations currently have little influence on choice of AT by either P&Cs or HCPs and other factors, such as usability, may be regarded by them as more important. Awareness of the evidence may also be a factor. All HCPs who used CIMT and robots and most (94%) who used FES thought that those ATs were evidence-based; yet only CIMT is recommended by both the UK and EBRSR guidelines.

These findings highlight the need to improve the evidence-base for ATs, particularly those that are currently being used and that satisfy the requirements of the respondents such as: ease of set up; fun to use; safe; comfortable and durable.

Mismatch between strength of research evidence and clinical use has been reported previously [34] and the survey identified examples of technologies that are becoming widely used in clinical practice for which there has been strong commercial drive and investment. For example, the Saeboflex ( http://www.saebo.com) is used in many UK centres, Bioness Inc. ( http://www.Bioness.com) who manufacture upper and lower limb FES devices report increasing sales and reimbursement, and O_2_ have launched a project to make the ‘Wii fit’ available to stroke patients as part of their Global e-health strategy ( http://www.oz.com). None of these devices has undergone large-scale clinical trials.

Conflict between key sources of information regarding effectiveness suggests that other factors are important in determining clinical use of ATs. Inevitably, in the absence of clear research evidence and guidance, HCPs’ decision-making is influenced by anecdotal evidence and their own experience. These may incorporate a complex integration of factors including observed clinical effectiveness, what they rate as important and the views of the patient and sometimes the carer. Our findings suggest that therapists take a pragmatic view when it comes to using ATs. They prioritise evidence, but also acknowledge that usability is crucial to avoid AT abandonment. One reason for this, that has been suggested, is lack of consideration of user opinion in design and selection of ATs [36].

Cost effectiveness is a key factor in the adoption of any new treatment into clinical practice. The cost-effectiveness of using robots for upper limb rehabilitation, for example, has been reported in a single randomised controlled trial that also demonstrated modest clinical benefits compared with usual care at 36 weeks [37], but further empirical research using economic analysis is required to demonstrate consistent results. In the UK a 3-arm RCT of robot therapy with an expected sample size of N = 720 is currently being undertaken ( http://www.nets.nihr.ac.uk/projects/hta/112605). The trial will generate the required evidence, however, cost-effectiveness in the use of expensive technologies is heavily dependent on how much devices are used and the proportion of patients who benefit. Our survey has shown that cost-effectiveness does not guarantee that new treatments, especially those that rely on a change in practice (such as ATs), will automatically be adopted [8].

### Opportunities for ATs in clinical practice

Changes in care pathways following stroke and the drive for more efficient use of rehabilitation services provide an opportunity to develop ATs, especially those that can be used outside hospital and without supervision. The results of the survey provide clear indicators for what is needed to enable this to happen: i.e. more usable technologies and better clinical evidence. Our findings provide indications for the design of new ATs. The top five factors considered by both HCPs and P&Cs as critical for acceptance were: evidence-based, ease of set-up, safety, comfort and durability. They also need to be generalizable to many patients at different stages of recovery and in different settings – factors that were shown to be met by some ATs. Knowledge of these factors and response to them is critical in the design of future ATs.

### Barriers to clinical use

The survey has identified barriers to clinical use of ATs, but understanding them is fundamental to overcoming them. Much can be learnt from the wider field. For example, Reynaud (2008), proposed different models of acceptance (an attitude) and adoption (a process) of technologies from the fields of information systems and sociology [38]. These models can be applied to any technology type, but can also be specialised to individual technologies. Acceptance models, such as the Technology Acceptance Model (TAM) [39,40], suggests that when users are presented with a new technology a number of factors influence their decision about how and when they will use it, including “perceived usefulness” (the extent to which a person believes that using a particular system would enhance their performance) and “perceived ease-of-use” (the extent to which a person believes that using a particular system would be free from effort). This model, which has been updated several times [41-43], corresponds with the findings from the survey presented in Table 4 for the high priority given by both HCPs and P&Cs to the ease of set up and use for an ideal technology. Technology adoption processes reported included those developed by Rogers [44] and Silverstone and Haddon [45]. In the former, the model aims to provide a framework for understanding how technology innovations change, and are changed, by their social contexts. In the latter, the model focuses mostly on the individual’s decision to buy or not to buy. At a systems level the Normalization Process model was developed to assist both service providers and research constituencies in understanding how health care interventions, technologies, and practices are implemented, embedded, and integrated in everyday life [46]. These models could be applied more widely within the AT field to assess key barriers to the translation of individual technologies and should be integrated into clinical effectiveness trials. Based on the survey’s findings, barriers reported by HPC and P&C were a lack of knowledge and access to ATs the following are recommendations to address these.

Knowledge-transfer and changes in service provision - key opportunities to translate ATs into clinical practice, are currently not exploited and therefore remain barriers. For example 41% of HCPs and 64% of P&Cs had not used ATs. When asked why not, 98/114 (86%) HCPs cited lack of access and 27/114 (24%), lack of knowledge. Among P&C respondents 48/78 (62%) knew nothing about them and 21/78 (27%) said they could not get one on the NHS. Similar responses were expressed in the ‘views’ section of the questionnaire, for example only 10% of P&Cs and 21% HCPs disagreed or strongly disagreed with the statement that it was ‘difficult to access training or advice’. Training was also identified as an important area for further development and could be used to raise HCPs awareness of research evidence.

Until ATs become a core element of therapist’s training they are unlikely to be used in routine clinical practice, but without evidence for their effectiveness, inclusion is hard to justify, despite the fact that many approaches to stroke rehabilitation currently taught are not evidence-based. A more pragmatic approach would be to include opportunities for learning about ATs (as well as training in using them) at post-qualifying level. Collaboration between universities, healthcare providers and the commercial sector may be an effective way of providing this. The wider study (of which this survey was part) began with an interactive exhibition of ATs that brought together commercial companies demonstrating a wide range of technologies, and patients, carers, researchers and clinicians. In doing so we increased clinicians’ awareness, knowledge and understanding of ATs and established a communication network between clinicians, researchers and the commercial sector that has been influential in the formation of the International Industrial Society in Advanced Rehabilitation Technologies (IISART). Awareness may be increased by similar exhibitions, run by trade organisations, in collaboration with universities providing healthcare courses nationally, or with regional or national specialist interest groups for HCPs. Keeping up-to-date with new information, given the time constraints in clinical practice, has been recognised as a barrier to achieving evidence-based stroke rehabilitation [47]. Professional bodies can play a role in removing barriers by providing independent advice, based on new evidence, and employing a variety of accessible Internet-based methods such as webinars and podcasts.

Access to ATs is posited as a key factor that influences whether therapists have used or prescribed them. Currently, access to ATs in the UK is through commissioners of stroke services, who require a business case to justify their provision that identifies the cost benefits of using ATs, both in the short and long term. This would ideally include service level benefits: proven implementation in other regions, a description of need, number of people suitable for treatment, level of resource required (e.g. reduced costs through fewer repeat visits to the service; reduced level of social care and reusability of equipment). It would also include plans for auditing use such as number of suitable patients and duration of use. In addition, commissioners need estimates of benefits for patients, such as quality of life outcomes and increased social activity. Increased collaboration between HCPs and manufacturers is an additional way of providing better access to ATs, through for example, training and ATs ‘on-loan’, enabling HCPs to be more knowledgeable and experienced in using them.

The survey identified a conflict between research evidence and clinical use of ATs, and thus a failure in translation of research into clinical practice. Translation may be more efficient if research studies are pragmatic – testing ATs in the environment in which they will be used clinically and incorporating robust examination of user’s views and the burden that the AT makes on users. Furthermore, involving end users, both HCPs and P&Cs, into the design of clinical trials, may facilitate translation.

### Characteristics of respondents that may have influenced the views expressed

P&Cs and HCPs had similar views about current upper limb post-stroke rehabilitation (although it is important to note that because P&Cs’ mean time post-stroke was 8 years their personal experience may not reflect current practice). Views on the use of ATs were also similar between groups, although P&Cs were more confident about using ATs at home than HCPs were about patients and carers using them. The views of P&Cs and HCPs regarding ATs are also remarkably similar, increasing the validity and usefulness of the findings for providers of stroke rehabilitation services, researchers and developers of ATs.

### Strengths and limitations of the study

The study has several strengths. It is based on a nationwide survey of a large number of HCPs (n = 292) who work with stroke in a range of settings and 123 P&C who have experienced a stroke. The questions were developed using data generated by focus groups and subsequently pre and pilot tested to ensure relevance, comprehensiveness and to minimise bias. However there were limitations. There is a selection bias in the way the questionnaires were designed and potentially responded to. The methodology was designed to recruit a sample of the population of P&Cs and HCPs working in stroke rehabilitation. However, it is likely that, being a self-selected sample, the results are biased towards the views of people interested in ATs. Factors that will have contributed to this are that some respondents would have attended the exhibition. Whilst the questionnaire data were from a national sample, the majority of whom had not attended the exhibition, and as such were likely to be more representative of national views and experiences, people may have been more likely to look for and complete the questionnaire if they had an interest in ATs. In that respect knowledge and use of ATs reported here might be greater than in the population as a whole and the views expressed may be more positive. Additionally, the response rate cannot be specified for the online version of the questionnaire, and the fact the data were combined with the paper version raises issues about whether the data are directly comparable. The number of individuals involved is uncertain, for instance we cannot guarantee that individuals have not completed more than one questionnaire, and the representativeness of the responses is uncertain. Motivation to respond to the questionnaire may have differed across the different professional groups, leading to biased estimates of the popularity of some interventions. Another limitation of the study is that it was based on self-report: HCPs reports of their practice may not reflect actual practices. Some patients had suffered their first stroke many years before completing the questionnaire; their experiences may therefore not be up-to-date. Moreover, a social desirability bias (adapting responses to meet what people believe they should be thinking) cannot be ruled out especially when considering attitudes and opinions. Test re-test reliability and internal consistency were not tested, which along with cognitive interviewing processes would have strengthened the data.

### Future work

This work has been conducted in parallel with surveys of stroke services to determine current AT rehabilitation methods and systematic literature reviews to provide both narrative descriptions and quantitative comparisons of each AT for upper limb function. This information will be used to determine which combination of ATs has the greatest probability of significantly improving upper limb rehabilitation following stroke, is cost effective and acceptable for use by patients and in health services. Results from this work have directly informed the design of a clinical trial which will be used to propose a new service delivery model, as well as provide an operational framework for future studies. Engagement with all stakeholders has been embedded through the whole programme of work.

Further work could explore the results of the current survey with, for example, qualitative interviews, with a purposive sample of respondents. Longitudinal studies would help to determine how quickly attitudes to arm and hand rehabilitation, and use of ATs in practice are changing.

## Conclusion

Research evidence was seen as critical to clinical use, yet our findings suggest that key barriers to the translation of ATs into clinical practice are usability, knowledge, education, awareness and access to ATs. Use of ATs and perceptions varied between professional groups as well as with years’ experience, but overall responses from HCPs & P&Cs were remarkably similar. Opportunities exist to overcome barriers, through better communication between manufacturers, educators, HCPs and P&Cs. With an understanding of these factors, AT designers, manufacturers and marketing experts can create ATs for HCP and P&C preferences and target marketing information more effectively. Based on our findings, improvements in AT design, pragmatic clinical evaluation, better knowledge and awareness and improvement in provision of services will enable useful ATs to be used in clinical practice and therefore contribute to better and cost-effective upper limb stroke rehabilitation.

## Competing interests

Jane Burridge – Scientific Advisory Board Hocoma A/T, previous consultancy for Ottobock and Bioness. Ian Swain - Director and shareholder in Odstock Medical. All other authors have no competing interests.

## Authors’ contributions

AMH co-designed the questionnaire, and data collection for the HCP questionnaire, performed the analysis and drafted the paper. JHB conceived of the study, co-ordinated the work, reviewed the questionnaire design, and refined the paper. SHD co-designed the questionnaire, reviewed the questionnaire design and commented on the paper. CSEH conceived of the study, reviewed the questionnaire design and commented on the paper. CM undertook data collection for the P&C questionnaire, performed the analysis and commented on the paper. LTT undertook data collection for the HCP questionnaire and commented on the paper. RT undertook data collection for the P&C questionnaire and commented on the paper. IDS conceived of the study, co-ordinated the work, reviewed the questionnaire design, and refined the paper. All authors read and approved the final manuscript.

## Pre-publication history

The pre-publication history for this paper can be accessed here:

http://www.biomedcentral.com/1472-6963/14/124/prepub

## Supplementary Material

Additional file 1 HCP questionnaire. Click here for file

Additional file 2 P&C questionnaire. Click here for file
