# Supplementary material for: Translation of evidence-based Assistive Technologies into stroke rehabilitation: users’ perceptions of the barriers and opportunities
Source: BMC Health Serv Res. 2014 Mar 12;14:124. doi: 10.1186/1472-6963-14-124 (PMC4007558; doi:10.1186/1472-6963-14-124)
Supplement: Additional file 1 — HCP questionnaire. [file 1472-6963-14-124-S1.doc]

Thank you for taking part in the ATRAS project by completing this questionnaire.

Before you begin you may find it helpful to know a little more about the project and the Questionnaire. Assistive Technologies (ATs) are electrical or mechanical devices designed to help people recover movement. They are normally used to augment conventional physiotherapy and are being used increasingly both in the UK and world-wide. In this project we are only interested in the use of ATs with people who have had a stroke and to help them use their arm and hand again.

Some examples of these are:

- Virtual reality – computer games that you play by moving your arm and hand. Sometimes you see an image of your arm and the way it is moving on the computer screen
- Dynamic splints e.g. Saeboflex – Theses are devices which you wear. They may have strapping or springs to help you open your hand to pick up objects
- Biofeedback - Where the device tells you immediately about your arm movement.
- Robots – Devices that support your arm and / or hand and help you to move while you either play computer games or practise manual tasks
- Constraint induced movement therapy - where you wear a glove on your “good hand” for about six hours a day for a few weeks
- Functional Electrical Stimulation – where you put sticky pads or a device over your skin which transmit small electrical impulses. This is often used to help you pick things up

The aim of the ATRAS project is to identify which ATs are most useful by looking at research evidence and consulting with users. The most promising ATs will then be tested in a large clinical trial and the findings will be used to improve the care-pathway for stroke rehabilitation across the NHS.

We are very grateful for your contribution.

Yours sincerely,

Prof Jane Burridge

Before you complete the questionnaire, please read the participant information sheet relating to this study. If you have any questions about the study please contact Dr A. Hughes on: 023 8059 5191 or [A.Hughes@soton.ac.uk](mailto:A.Hughes@soton.ac.uk). When you are happy to continue, please read the information below and then begin the questionnaire.

**1. All responses are anonymous.**

**2. Participation is voluntary. It is up to you whether or not to complete the questionnaire. You can also decide not to answer particular questions if you choose.
3. By completing the questionnaire, you are giving us consent to use the information supplied for research purposes.
4. We are interested in your opinions. There are no right or wrong answers. Please try to provide completely open and honest answers. Most of the questions will require you to shade the one circle per question which most accurately reflects your own opinion using a BLUE or BLACK ball-point, like this . 5. If you make a mistake, do not worry. Just cross through the answer you DO NOT want and make your selection as before.**

**6. Although some questions appear similar, there are differences between them. Please treat each one as a separate question.**

**It should only take you 10-15 minutes to complete the** questionnaire.

| **SECTION A: ABOUT YOU** | **ABOUT ATs YOU HAVE USED/PRESCRIBED** |
| --- | --- |
| 1. What is your profession ?  Nurse or Matron  OT  PT  Doctor  Other (please write which one below)  ……………………………………………  2. How many years have you worked with people with stroke?    3. Which group /s of people with stroke do you primarily work with / have you worked with ? (please shade **up to two** options)  NHS - Acute (fewer than 4 weeks post stroke)  NHS - Sub acute (from 4 weeks to 6 months post stroke)  NHS - Chronic (over 6 months post stroke)  Private practice  4. If you have had information on ATs where did you get this? (Shade as many as appropriate)  Internet  Hospital  Company  Courses  Continuing professional development training  Undergraduate Courses  Other (please write where below)  …………………………………………..  I have not had any information on ATs  5. Have you ever used / prescribed ATs for your patients’ arms  Often go to question 7  Sometimes go to question 7  Never go to question 6  6. If never, why haven’t you used / prescribed them (answer as many as necessary)  Do not think they work  Do not know anything about ATs  Do not have access to any  **Go to question 10, section B, page 3** | 7. Which of the following ATs have you used / prescribed to help with hand or arm therapy? (shade all appropriate)  Virtual reality e.g.Wii or other computer games  Dynamic splints e.g. Saeboflex  Biofeedback  Robots  Constraint Induced Movement Therapy  Functional Electrical Stimulation  Other - If other please write which ones  …………………………………………..  8. Which of the following ATs have you used/prescribed **most frequently** to help with hand or arm therapy? (please shade **only one** option)  Virtual reality e.g.Wii or other computer games  Dynamic splints e.g. Saeboflex  Biofeedback  Robots  Constraint Induced Movement Therapy  Functional Electrical Stimulation  Other (please write which one below)  …………………………………………….  9. Thinking about the AT you identified in question 8, do you think this device….   |  | Yes | No | Don’t know | | --- | --- | --- | --- | | is easy to set up and use? |  |  |  | | should primarily be used in therapy sessions? |  |  |  | | looks good? |  |  |  | | is durable and reliable (did not break down)? |  |  |  | | is comfortable to use? |  |  |  | | has a low risk of harm? |  |  |  | | is based on scientific evidence? |  |  |  | | is good value for money? |  |  |  | | is suitable to be used by patients at home? |  |  |  | | is fun to use? |  |  |  | |

| **SECTION B: YOUR BELIEFS ABOUT ATS AND ARM REHABILITATION** | | | | | | |
| --- | --- | --- | --- | --- | --- | --- |
|  |  | ***Strongly disagree*** | ***Disagree*** | ***Neutral*** | ***Agree*** | ***Strongly agree*** |
| 10. | In my experience, people with stroke get good therapy for the arm and hand while in hospital |  |  |  |  |  |
| 11. | In my experience, people with stroke get good therapy for the arm and hand when they leave hospital |  |  |  |  |  |
| 12. | I think that ATs will be useful in the future but need more development |  |  |  |  |  |
| 13. | Carer support is important for using ATs successfully |  |  |  |  |  |
| 14. | It is difficult for me to get adequate information, training or advice in using/prescribing ATs |  |  |  |  |  |
| 15. | I am concerned that ATs may be used to replace skilled therapy |  |  |  |  |  |
| 16. | Using ATs in rehabilitation is an efficient use of therapy time and resources |  |  |  |  |  |
| 17. | I do not know much about ATs |  |  |  |  |  |
| 18. | To my knowledge, there is no reason why ATs should not be used in the first 4 weeks after stroke |  |  |  |  |  |
| 19. | ATs should only be used when conventional therapy has failed |  |  |  |  |  |
| 20. | ATs should only be used on the recommendation of a healthcare professional |  |  |  |  |  |
| 21. | ATs should only be used with a healthcare professional present |  |  |  |  |  |
| 22. | Unsupervised use of ATs may encourage abnormal or poor movement |  |  |  |  |  |
| 23. | People with stroke lack the confidence to use ATs at home |  |  |  |  |  |

| **SECTION C: IDEAL DESIGN** |
| --- |

**24. Imagine you are designing the 'ideal' AT. Please put the following factors in order of importance to you (1 is the most important and 10 is the least important). Use each number only once**

|  | Example |  |
| --- | --- | --- |
| Fun | 1 |  |
| Good research evidence | 5 |  |
| For use mainly under the supervision of a healthcare professional | 9 |  |
| For use mainly unsupervised at home | 7 |  |
| Comfortable | 3 |  |
| Attractive appearance | 2 |  |
| Value for money | 6 |  |
| Easy to set up and use | 4 |  |
| Durable and reliable (does not break down) | 10 |  |
| Low risk of harm | 8 |  |

|  |
| --- |
| **THANK YOU FOR TAKING PART IN THIS RESEARCH**  **We have tried to ensure that the questions do not cause any distress to you. However, if completion of the questionnaire has raised any upsetting concerns, you may wish to discuss these with your GP.**  **NOW PLEASE RETURN THE COMPLETED QUESTIONNAIRE IN THE FREEPOST ENVELOPE PROVIDED** |

| **IF YOU HAVE ANY QUESTIONS PLEASE CONTACT:**  **Dr Ann-Marie Hughes PhD MCSP, Faculty of Health Sciences, University of Southampton**  **Highfield, Southampton, SO17 1BJ. Tel: 023 8059 5191 Email: A.Hughes@soton.ac.uk** |
| --- |
